# *CsTT8* regulates anthocyanin accumulation in blood orange through alternative splicing transcription

**DOI:** 10.1093/hr/uhad190

**Published:** 2023-09-19

**Authors:** Jianhui Wang, Rui Xu, Shuangping Qiu, Weichun Wang, Fan Zheng

**Affiliations:** Department of Food Science and Engineering, School of Food and Biological Engineering, Chengdu University, Chengdu 610106, China; Zhang Lan Honors College, Chengdu University, Chengdu 610106, China; Department of Food Science and Engineering, School of Food and Biological Engineering, Chengdu University, Chengdu 610106, China; Department of Food Science and Engineering, School of Food and Biological Engineering, Chengdu University, Chengdu 610106, China; Department of Food Science and Engineering, School of Food and Biological Engineering, Chengdu University, Chengdu 610106, China; Department of Food Science and Engineering, School of Food and Biological Engineering, Chengdu University, Chengdu 610106, China; Zhang Lan Honors College, Chengdu University, Chengdu 610106, China

## Abstract

A homologous gene of basic-helix–loop–helix *AtTT8* in *Arabidopsis thaliana* was identified in juice sac cells of pulp tissues from blood orange (*Citrus sinensis* cv ‘Tarocco’), which was designated as *CsTT8* in this study. Additionally, the mRNA levels of *TT8* with the full-length open reading frame were significantly higher in ‘Tarocco’ than in mutant fruit lacking pigment in pulp or peel tissues. However, an alternative splicing transcript, Δ15-*TT8*, with the fourth exon skipped, was also identified from transcripts different in length from that in ‘Tarocco’. The mRNA levels of Δ15-*TT8* were higher in mutant fruit lacking pigment in pulp or peel tissues than in the wild type. Therefore, the *TT8*/Δ15-*TT8* mRNA level ratio was found to be crucial for sufficient pigment in either pulp or peel tissues. TT8 from blood orange fruit demonstrated the capacity for nucleus localization and binding to other proteins. In contrast, Δ15-TT8, lacking the fourth exon, lost its ability to interact with RUBY1 and to localize at the nucleus. Using a dual luciferase reporter assay and transient overexpression in tobacco, we proved that two regulatory complexes formed by a functional TT8 with different MYB(v-myb avian myeloblastosis viral oncogene homolog)-type partners significantly promoted expression of an anthocyanin biosynthetic gene and a proton pumping gene, leading to anthocyanin and citrate production. Our findings suggest that TT8, rather than dysfunctional Δ15-TT8, is possibly involved in modulating anthocyanin biosynthesis and its transport into vacuoles by proton gradients. However, increased mRNA levels of the dysfunctional alternative splicing transcript may act as a negative feedback to downregulate TT8 expression and limit anthocyanin accumulation in blood oranges.

## Introduction

The blood orange is a cultivar of sweet orange (*Citrus sinensis*), and anthocyanin was not only found in juice sac cells of pulp tissues but also distributed in peel tissues. In fruits, anthocyanins are a group of natural phenolic compounds responsible for coloration in pulp or peel tissues. They are glycosylated polyhydroxy and polymethoxy derivatives of flavylium salts. ‘Tarocco’ [*C. sinensis* (L.) Osbeck] is the most common and widespread blood orange variety across the world. Water-soluble pigments are scavengers of radical species. It was reported that, among the active pigments, cyanidin-3-glucoside and cyanidin 3-(6″-malonyl) glucoside are the main components in fruit juice from blood orange [1]. Cyanidin 3-glucoside has strong anti-oxidation effects and can improve inflammation and metabolic diseases associated with obesity. Due to the increasing interest in nutraceutical compounds in the food industry, the demand for fruit juice rich in pigments is increasing, and the consumption of drinking powders with flavonoid compounds from blood orange is developing rapidly in the Chinese market. At present, Sichuan province, southwestern China, has become the largest blood orange production area, with 22 000 hectares, and accounts for 86% of total yield in China. Better understanding of anthocyanin regulation in blood orange will therefore be useful for the improvement of cultivars and the intensive processing of fruits for citrus industry development.

Anthocyanin biosynthesis in the plant is a metabolic branch of the flavonoid synthesis pathway. A transposon was inserted into the promoter of an MYB-type transcription factor (designated RUBY1) to activate its own expression and then promote anthocyanin production in blood orange [2]. Anthocyanin biosynthesis induced by HY5 expression and upregulation of RUBY1 transcription by light were found in blood orange [3]. A TT8 transcription factor with a basic helix–loop–helix (bHLH)-type motif interacted with RUBY1 and WD40 to form an MBW (MYB-bHLH-WD40) complex and was involved in regulating anthocyanin biosynthesis in our previous studies [4]. AtTT8, AtEGL3, and AtGL3 with a bHLH-type conserved domain in *Arabidopsis thaliana* have similar expression patterns and redundant functions [5]. The members of the bHLH-type protein family possess a bHLH conserved domain containing a basic amino acid region at the N-terminal and an HLH region at the C-terminal. Compared with CsTT8 in blood orange, CgbHLH1, identified in a pumelo having peel tissues with a purple color [6], had higher identity with AtGL3 in *A. thaliana* at amino acid level.

Anthocyanin transport and storage markedly affect coloration in different plants. An acidified vacuolar environment will benefit anthocyanin storage in vacuoles in juice sac cells. AN1 in petunia contained a bHLH-type conserved domain at the C-terminal which regulated vacuolar acidity as well as anthocyanin production in petal cells [7]. PH4 of petunia is an R2R3 MYB-type protein that activates vacuolar acidification through interactions with the bHLH transcription factor AN1 [8]. AN11-AN1-PH4 protein complexes were formed and then bound to PH3 (WRKY transcription factor), which activated expression of proton pump gene *PH8* to facilitate vacuole acidification [9]. Different bHLH-type transcription factors were found in varieties of citrus, designated *NOEMI*, controlling acidity in fruit and proanthocyanidins in seed [10]. Disruption of expression of the bHLH-type transcription factor gene *CitAN1* and the MYB-type transcription factor gene *CitPH4* was associated with downregulation of expression of proton pump genes in high-pH citrus fruits [11]. In *Citrus*, the CsPH4-NOEMI regulatory complex contributes to the activation of proanthocyanidin biosynthetic genes, a major class of flavonoids, via a positive feedback loop [12].

Light and plant hormones induced expression of important transcription factors to produce anthocyanin in many fruit trees [13]. Citrus is one of the non-climacteric fruit trees and fruit quality development is mainly regulated by ABA signaling. During sweet orange fruit development and ripening, the expression pattern of some ABA receptors mirrored ABA accumulation, whereas that of *CsPP2CA* genes paralleled hormone accumulation, together modulating ABA perception, downstream signaling, and consequently physiological responses [14]. However, there is still a lack of clarity about the mechanism of the interplay between ABA content and other transcription factors, such as TT8, in blood orange fruit.

Alternative splicing is an important mechanism of post-transcriptional modification and dynamically regulates multiple physiological processes in plants under abiotic stress or at critical developmental stages. Post-transcriptional splicing of nascent RNA contributes to widespread intron retention in plants [15]. At present, the molecular mechanism by which these splicing factors are recruited and are then involved in alternative splicing remains unclear. In eukaryotes, genes are transcribed to produce mRNA precursors (pre-mRNA) in the nucleus, which are alternatively spliced to ultimately produce multiple different mature mRNAs, and this process can increase the diversity of gene products and inevitably affect the function of each protein. A total of 1133 genes associated with 1425 alternative splicing events in orange were identified [16]. Alternative splicing has played a major role in defining protein diversity, which could be linked to phenotypic changes under different biological or abiotic stresses. Alternative splicing of CiFD formed two different proteins (CiFDα and CiFDβ) in *Citrus*, out of which CiFDβ can form a more direct and simplified pathway, compared with CiFDα—one that is independent of the FAC complex—to regulate drought-induced flowering [17]. However, little is known so far about alternative splicing events contributing to fruit quality in fruit trees.

This study is the first to report a dysfunctional ΔTT8 produced by an alternative splicing event, which markedly affected anthocyanin biosynthesis and citrate production in blood orange fruit. On the contrary, TT8 with a full-length open reading frame, contributed to the formation of two independent complexes by binding with two different MYB-type partners, and then transactivated the expression of a flavanone hydroxylase gene and a proton pumping gene in the downstream pathway. Intriguingly, increasing mRNA levels from alternative splicing of Δ15-*TT8* may downregulate *TT8* expression and then affect pigment accumulation in blood orange fruit.

## Results

### Identification of alternative splicing events in *CsTT8*

A *CsTT8* with a full-length open reading frame identified in pulp tissues from ‘Tarocco’ fruit (homologous gene with identifier orange1.1g037798m in the ‘Valencia’ sweet orange database) was grouped with *A. thalianaAtTT8* (TRANSPARENT TESTA 8) into an independent cluster according to a phylogenetic tree in comparison with 194 bHLH motif DNA-binding superfamily proteins selected from sweet orange ‘Valencia’ in the ICGC database (Supplementary Data Fig. S1).

**Figure 1:**
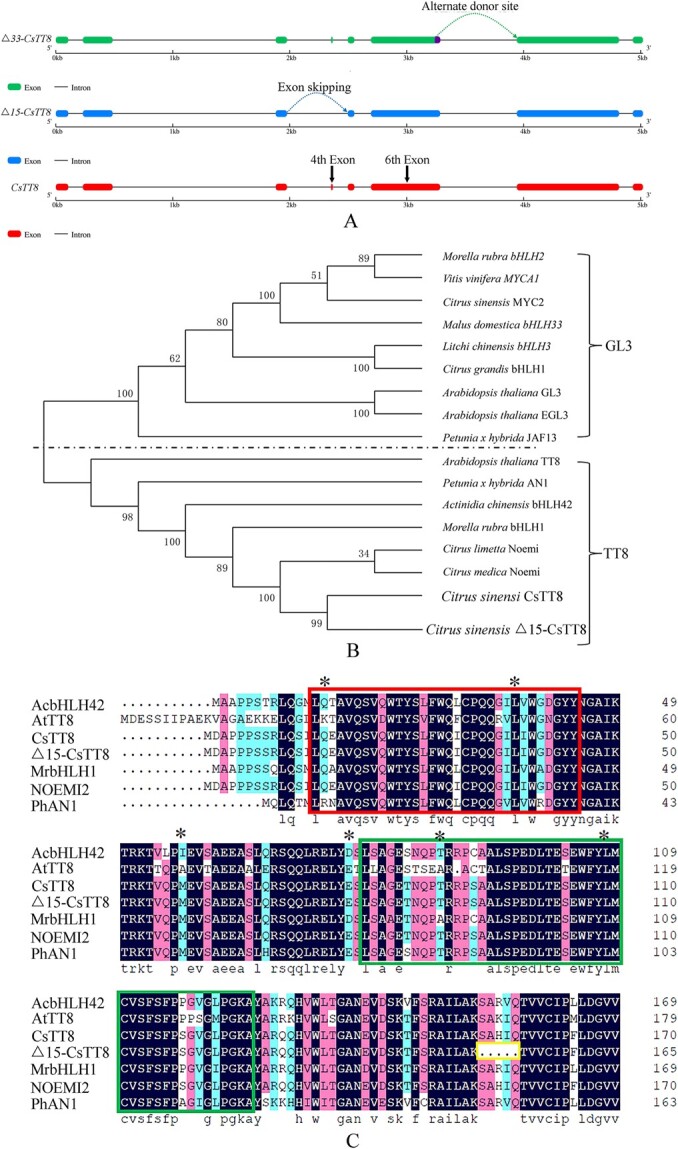
Alternative splicing transcripts of *TT8* isolated from blood orange and multiplex alignment of different bHLH-type proteins derived from fruit trees.

Three different alternative splicing events in *TT8* from ‘Tarocco’ fruit were observed (Fig. 1A). An alternative donor site event was found (Fig. 1A), out of which the 5′ terminal of sequences at the sixth exon was alternatively spliced and then designated as Δ33-*CsTT8*, with length 2035 bp. Moreover, an exon-skipping event was also identified, so that the fourth exon was alternatively spliced and then designated as Δ15-*CsTT8*, with length 2070 bp (Fig. 1A). Additionally, a full-length open reading frame of *TT8* was obtained with length 2085 bp. *CsTT8*-encoded proteins clustered together into the AtTT8 group (Fig. 1B). The fourth exon of *TT8* underwent alternative splicing through exon skipping. As a result, Δ15-TT8 lacked a conserved domain of the SAHIQ motif (Fig. 1C). Furthermore, an amino acid sequence (SSLQLPPSGSG) at the 5′ terminal of the sixth exon was absent, and the spliced fragment was in the non-conserved domain between MYC and the bHLH motif in Δ33-TT8 (data not shown). MrbHLH1 in Chinese bayberry, AcbHLH42 in kiwifruit and PhAN1 in petunia had higher similarity to TT8 in blood orange by 70.4, 65.5, and 62%, respectively, out of which MrbHLH1 and MrMYB1 were found to form a regulatory complex and to activate transcription of catalytic enzyme genes involved in the anthocyanin synthesis pathway [23]. However, the amino acid identity between CsTT8 isolated from *C. sinensis* and CgbHLH1 from *Citrus maxima* was only 35.7%, but CgbHLH1 was reported to contribute to anthocyanin production in purple-skin pomelo [6]. However, the physiological function of alternative splicing transcripts of *TT8* in blood orange fruit is still unknown.

**Figure 2:**
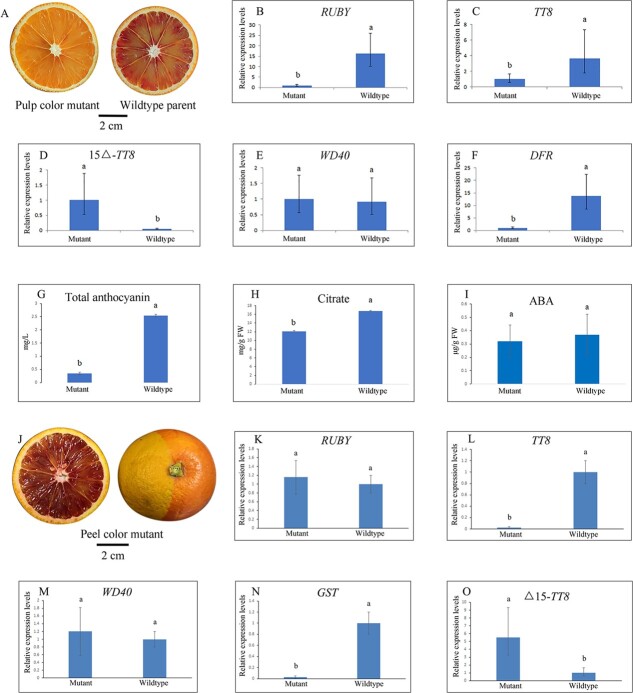
Comparative analysis of differentially expressed genes and fruit quality in comparison with wild type and mutant lacking sufficient pigment in pulp or peel tissues. **A** Mutant fruit lacking pigments in pulp tissues. **B**–**F** mRNA levels of RUBY1 (**B**), TT8 (**C**), Δ15-TT8 (**D**), WD40 (**E**), and DFR (**F**). **G** Total anthocyanin content. **H** Citrate content. **I** ABA content. **J** Fruit of chimera lacking pigment in half of peel tissue. **K**–**O** mRNA levels of *RUBY* (**K**), *TT8* (**L**), *WD40* (**M**), GST (**N**), and Δ15-*TT8* (**O**).

In our preliminary study, nanopore sequencing technology was performed to generate long-read RNA-seq data in comparison with mutant fruit lacking pigment in pulp tissues and the wild type. Then ASTALAVISTA software (http://genome.imim.es/astalavista) was used to characterize alternative splicing events for whole transcriptome data from reference annotations. Furthermore, percent spliced-in (PSI) values are commonly used to report alternative pre-mRNA splicing changes between different samples. Using PSI-Sigma software (https://github.com/wososa/PSI-Sigma), alternative splicing changes for Δ15-TT8 in the mutant were estimated as 7.82% with ΔPSI (adjusted *P* value <.05), which was significantly higher than for other alternative splicing transcripts. Therefore, investigation of the differential expression of Δ15-TT8 and its functions have priority over other transcripts in the following studies.

### Differential expression of *TT8* from mutants lacking pigment in pulp or peel tissues

mRNA levels of *RUBY1* and *DFR* in pulp tissues of the wild type were significantly higher than those from the mutant lacking sufficient pigment in pulp tissues (Fig. 2B and F). However, the relative expression level of *WD40* in the wild type did not significantly differ from that in the mutant. Notably, the wild-type fruit exhibited a higher expression level of *TT8* in pulp tissues than the mutant. Intriguingly, the relative expression level of alternative splicing Δ15-*TT8* was significantly lower in the wild type than a mutant lacking pigment in pulp tissues (*P* <.05). Additionally, the mutant had less anthocyanin content in pulp tissues (0.35 mg/l) than the wild type (2.55 mg/l; *P* < .05). Citrate content in the wild type was 16.76 mg/g FW, which was significantly higher than in the mutant (*P* < .05). The ABA content was 0.369 μg/g FW in the wild type and 0.32 μg/g FW in the mutant (*P* >.05).

**Figure 3:**
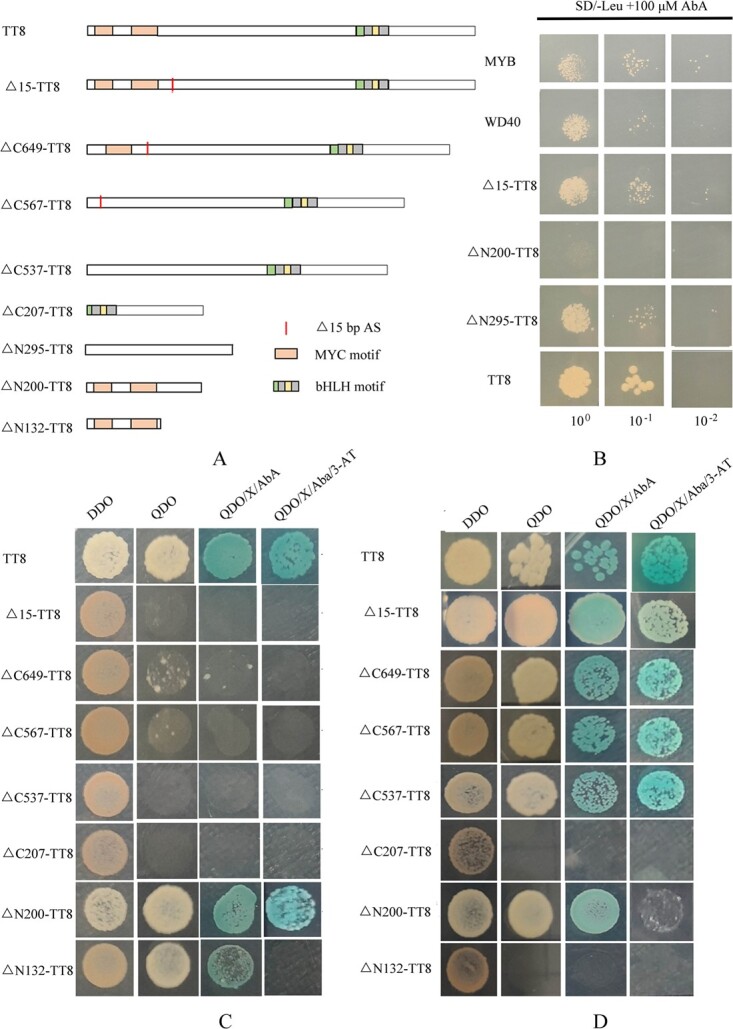
*In vitro* yeast hybrid of TT8 and its truncated proteins with others. **A** Many different truncated Δ − TT8 proteins constructed by partial-length open reading frame sequences of *TT8*. **B** Truncated Δ − TT8 proteins interacted with the promoter sequence of *F3′H* in the yeast one-hybrid assay. **C** Truncated Δ − TT8 proteins interacted with RUBY1 in the yeast two-hybrid assay. **D** Truncated Δ − TT8 proteins interacted with WD40 in the yeast two-hybrid assay.

By chance, a chimera fruit with yellow color in one half and red color in the other half of the peel tissue was observed in the same orchard. However, obvious visible pigments in the pulp tissues were evenly distributed after peeling. Therefore, each part of the peel tissues from a chimera fruit was used for RNA quantitative analysis; however, anthocyanin measurements were not performed due to the small quantity of peel tissue from this chimera fruit (Fig. 2J). Results indicated that the mRNA levels of *RUBY1*, *TT8*, and *GST* were higher in pulp tissue-specific compared with flavedo (data not shown). Interestingly, the relative expression levels of Δ15-*TT8* in yellow peel tissues lacking sufficient pigment was significantly higher than in the red part of the peel (*P* < .05). Furthermore, mRNA levels of *TT8* with a full-length open reading frame were significantly higher in the red-colored part of the peel tissues than in the yellow-colored part of the peel (*P* < .05). This suggests that the *TT8*/Δ15-*TT8* ratio of mRNA levels may influence anthocyanin accumulation in the pulp or peel tissues of blood orange, which means that a higher ratio of *TT8*/Δ15-*TT8* may promote more anthocyanin production in the fruit. To gain a better understanding of the mechanisms regulating anthocyanin biosynthesis in blood orange, molecular biology studies of *TT8* with a full-length open reading frame and Δ15-*TT8* lacking the fourth exon were performed in the following experiments.

**Figure 4:**
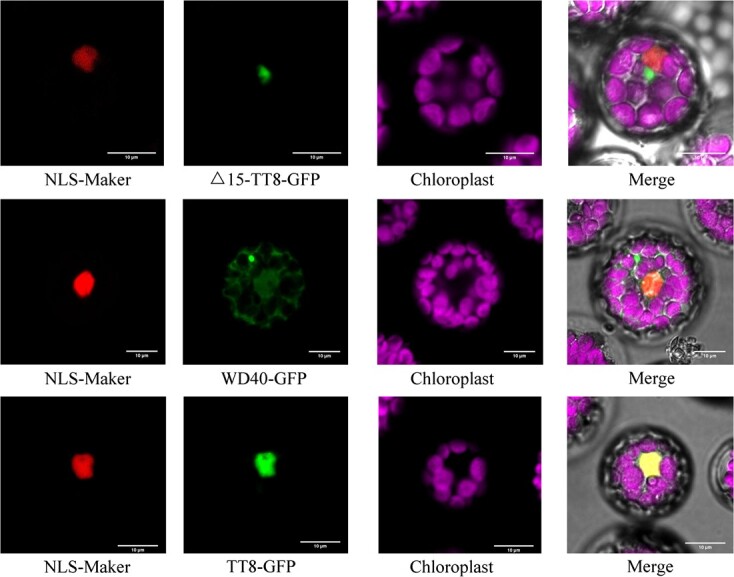
Nucleus co-localization analysis in *Arabidopsis thaliana* protoplasts by transient expression of TT8 and others.

### T‌T8 interactions with proteins and nucleic acid sequences involved in anthocyanin biosynthesis

Many different truncated Δ-TT8 proteins were constructed with partial lengths of open reading frame sequences for *TT8*. Among these, Δ15-TT8, which lacked the fourth exon, was found in ‘Tarocco’ fruit (Fig. 3A). In the yeast one-hybrid assay, RUBY1, WD40, and TT8 were found to bind to the *F3′H* promoter (Fig. 3B). However, ΔN200-TT8 protein was unable to bind to the promoter region, indicating that a domain closed to the bHLH motif at the C-terminal of TT8 plays a crucial role in interactions with nucleic acid sequences of anthocyanin biosynthetic genes.

In the yeast two-hybrid assay, it was observed that both TT8 and ΔN200-TT8 co-transformed by RUBY1 were able to grow on QDO medium with X-α-Gal and 10 mM 3-AT (Fig. 3C). This suggests that the SAHIQ motif encoded by the fourth exon of *CsTT8* is essential for the binding of TT8 to RUBY1 in blood orange fruit. Furthermore, ΔN207-TT8 and ΔN132-TT8 were unable to bind to co-transformed WD40, indicating that a fragment of amino acid sequences between MYC and the bHLH motif in TT8 significantly affects its interactions with WD40 (Fig. 3D). The alternative splicing transcript Δ15-TT8, found in ‘Tarocco’, has lost its ability to interact with RUBY1, although Δ15-TT8 was able to bind to the promoter region of *F3′H*. Hence, a fragment of the SAHIQ motif encoded by the fourth exon of *TT8* plays a crucial role in protein complex formation by binding to RUBY1.

### Subcellular localization of TT8

After DAPI staining, the cell nucleus of *Nicotiana benthamiana* was visualized using a red fluorescence signal, and then the EGFP target protein with a green fluorescence signal was also observed by laser confocal microscopy under 20× magnification (Supplementary Data Fig. 2). Therefore, the green fluorescence emitted from EGFP-Δ15-TT8 or EGFP-WD40 and the red signal from the nucleus after DAPI staining did not merge at a specific position, indicating that Δ15-TT8 or WD40 did not localize at the cell nucleus. However, red fluorescence from the cell nucleus merged with the green fluorescence signal from EGFP-TT8 or EGFP-RUBY1 can produce an overlying signal in yellow. Therefore, TT8 and RUBY1 were located in the cell nucleus, as observed in a preliminary experiment.

Consequently, red fluorescence was used to visualize nuclear co-localization sequences (NLS peptides) as a marker in the *Arabidopsis* protoplast system. This was performed to further verify the localization of TT8 in the nucleus. Results showed that Δ15-TT8 and WD40 were still located outside the cell nucleus (Fig. 4B). However, when the full-length open reading sequence of TT8 was present it co-localized with the NLS marker’s signal in the cell nucleus. A subcellular localization assay found that Δ15-TT8 lost the ability of nucleus localization, while TT8 was able to enter the nucleus and then regulate gene expression through a protein complex.

### T‌T8 transcriptional activation of expression of downstream genes through two different regulatory complexes

**Figure 5:**
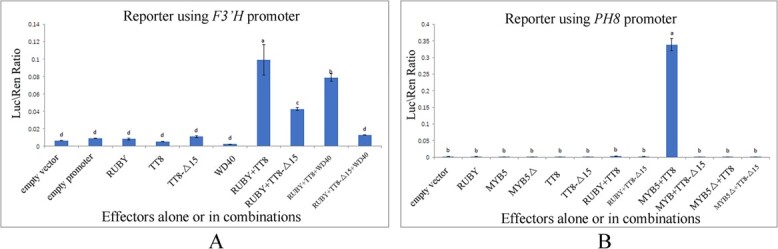
Dual luciferase reporter assay of transcription activation for TT8.

A combination of TT8 and RUBY1 effectors effectively transactivated the expression of a reporter with a promoter from an anthocyanin biosynthetic gene, *F3′H*, resulting in the highest luciferase ratio (LUC/REN, 0.099) compared with an effector alone or combinations of other effectors (*P* < .05, Fig. 5A). Additionally, the second highest LUC/REN ratio (0.079) was observed in combinations of RUBY1, TT8, and WD40 effectors. However, a lower LUC/REN ratio (0.042) was measured in combinations of Δ15-TT8 and RUBY1 effectors. These observation of luciferase changes suggest that TT8 and its partners form a protein complex to transactivate gene expression, but the transactivation activity of Δ15-TT8 was severely weakened.

In another dual luciferase reporter assay, a combination of TT8 from blood orange and MYB5 (an MYB-type transcription factor) from sweet orange was able to transactivate expression of the reporter with a promoter from the proton pump gene *PH8* with the highest LUC/REN ratio (0.34), significantly higher than with an effector alone or in combinations of other effectors (*P* < .05, Fig. 5B). However, a lower luciferase LUC/REN ratio (0.0014) was produced with the combination of Δ15-TT8 and MYB5. A lower luciferase ratio was also produced with the combination of TT8 and ΔMYB5 (alternative splicing transcript). It is possible to hypothesize that TT8 bound to RUBY1 or MYB5 to form two different regulatory complexes, which modulate anthocyanin biosynthesis and vacuole acidification, respectively. However, Δ15-TT8 partially or completely lost the function of transcription activation of the expression of downstream genes.

### A regulatory complex formed by TT8, RUBY1, and WD40 regulates different biosynthetic pathways

**Figure 6:**
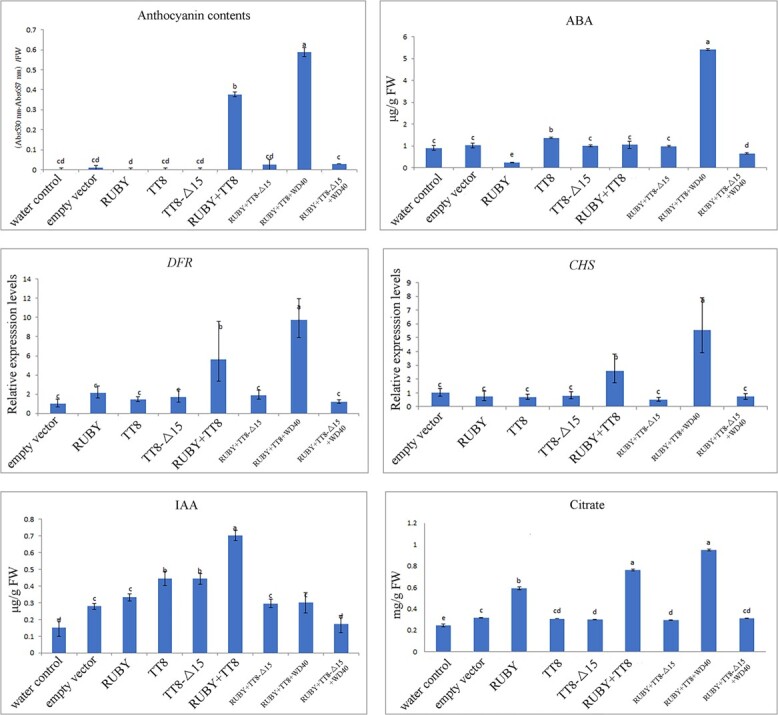
Transient overexpression in tabacco infiltrated with combinations of TT8 and others.

In a transient overexpression experiment (Supplementary Data Fig. S3), purple-gray-colored leaves were observed in tobacco (Fig. 6A). Extracts from these leaves infiltrated with a combination of TT8, RUBY1, and WD40 were obtained, resulting in anthocyanin production of 0.59 (OD_530_–OD_651_) 100 mg/g FW, which was higher than TT8 alone or in combination with other partners (Fig. 6B). In comparison, infiltration with a combination of non-functioning Δ15-TT8 and RUBY resulted in lower anthocyanin production, with 0.03 (OD_530_–OD_651_) 100 mg/g FW. Consequently, chalcone synthase (*CHS*) and dihydroflavanol 4-reductase (*DFR*) genes, which are involved in the anthocyanin biosynthetic pathway, showed higher relative expression levels in leaves infiltrated with a combination of TT8, RUBY1, and WD40 compared with Δ15-TT8 alone or in combination with its partners (*P* < .05).

Moreover, leaves that were inoculated with a combination of TT8, RUBY1, and WD40 exhibited citrate production of 0.949 mg/g FW, which was significantly higher compared with those inoculated with Δ15-TT8 alone or in combination with its partners (Fig. 6B; *P* < .05). Additionally, ABA contents were also higher in leaves infiltrated with combinations of TT8, RUBY1, and WD40, which were higher than in those infiltrated with Δ15-TT8 alone or in combination with its partners (*P* < .05). However, IAA contents in leaves infiltrated with TT8 alone or in combination with its partners did not exhibit consistency with ABA levels across each treatment.

In a transient overexpression experiment, TT8 bound to RUBY1 and WD40 to form an MBW (MYB-bHLH-WD40) complex, which effectively induced anthocyanin and citrate production in the inoculated leaves of tobacco. Interestingly, it is worth noting that an MBW regulatory complex might activate the ABA biosynthetic pathway. However, nether Δ15-TT8 alone nor its combinations with its partners effectively promote higher anthocyanin, citrate or ABA production in tobacco. Thus, a higher ABA/IAA ratio was found in leaves inoculated with the MBW regulatory complex to produce more anthocyanins. *In accordance with, the levels of ABA contents was increased rapidly in juice sac of sweet orange, but the trend curves of IAA contents in juice sac are shown as inverse V shapes across fruit development* [24]. Taking these results together, a regulatory complex formed by TT8, RUBY1, and WD40 in blood orange possibly plays important roles in versatile metabolic pathways, including secondary metabolites and plant hormone biosynthesis.

### Effect of exogenous ABA on fruit quality

**Figure 7:**
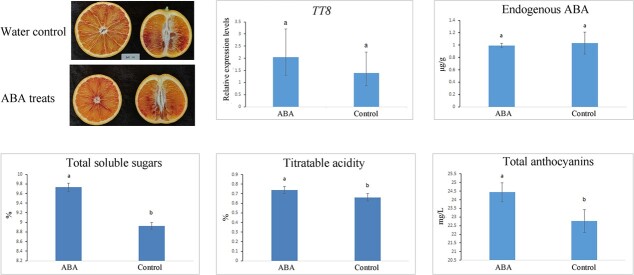
Quality analysis of fruits harvested from trees treated by exogenous ABA spraying and controls sprayed with water.

After a 3-month period of exogenous ABA application on fruit trees, total anthocyanin content in fruit was 24.43 mg/l, which was significantly higher than the 22.76 mg/l found in controls at the fruit ripening stage (*P* < .05; Fig. 7). Moreover, the titratable acidity in fruit treated with exogenous ABA was 0.73%, which was higher than the 0.66% in controls (*P* < .05). However, the endogenous ABA content in fruits was not significantly different between ABA-treated and control fruits at the fruit ripening stage (*P* > .05). After a month of spraying the tree with ABA, the relative expression levels of *TT8* in fruits was higher than in the control group (*P* > 0.05; Fig. 7). In conclusion, application of exogenous ABA to fruit trees may upregulate *TT8* transcription before fruit ripening, thereby improving fruit quality.

## Discussion

The time-course transcriptomic analysis using RNA-seq technology in blood orange revealed a complex, dynamic gene expression profile during fruit developmental and ripening periods [25]. Besides, mature mRNA via alternative splicing is an important mechanism of post-transcriptional modifications and dynamically regulates versatile physiological functions in plants. Recently, many studies have been reported about key transcription factor modifications regulating flowering through alternative splicing events in plants as affected by diurnal rhythm [26], temperature [27], and the abiotic environment [28]. Early flowering is a critical adaptive trait that strongly influences reproductive fitness in a short growing season. Moreover, a novel transcript of *chitinase* to produce defense-related acidic chitinase II was specifically induced by alternative splicing in *Citrus clementina* leaves infested by the two-spotted spider mite [29]. In this study, it is possible to hypothesize that TT8 alternative splicing might be triggered to balance anthocyanin accumulation in blood orange fruit before the ripening stage under low-temperature stress by an unknown mechanism.

Both exon skipping and alternative donor site events were identified in juice sac cells from ‘Tarocco’. Exon skipping events resulted in a transcript of Δ15-*TT8*, which lacked the conserved motif encoded by the fourth exon of the nearby MYC domain. Besides, the alternative donor site event generated another transcript of Δ33-*TT8*, which lacked a partial-length sequence at the end of the sixth exon. Based on differentially expressed genes in mutant fruit lacking pigment in pulp or peel tissues, the transcription ratio of *TT8*/Δ15-*TT8* might determine the sufficient production of anthocyanin in either pulp or peel tissues. Overall, alternative splicing transcript Δ15-*TT8* negatively regulates its expression in blood orange fruit through a feedback mechanism to balance the anthocyanin in juice sac cells.

**Figure 8:**
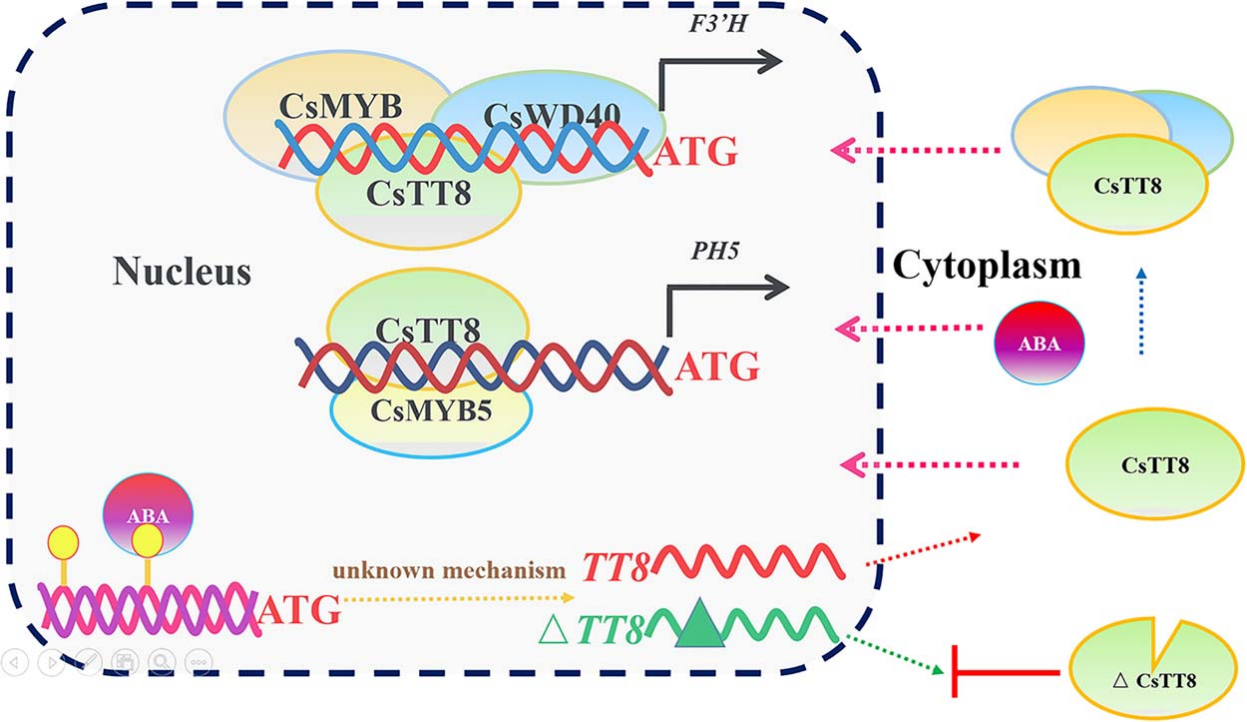
Model synergistic regulation of anthocyanin biosynthesis and vacuole acidification by TT8.

In this study, a novel model proposing the synergistic regulation of versatile physiological functions by TT8 in blood orange during fruit ripening is presented (Fig. 8). During fruit development and ripening, more ABA or other stimulators pass through the nuclear envelope and promote expression of *TT8* at the nucleus. However, the mechanism of multiple spliced mRNAs produced in the nucleus or cytosol remains unknown. Different proteins are produced by alternative splicing transcripts of *TT8*. TT8 with the full-length open reading frame can pass through the nuclear envelope and bind to RUBY1 and WD40. On the contrary, Δ15-TT8, lacking the SAHIQ motif, is unable to enter the nucleus and to bind to RUBY1. The SAHIQ motif encoded by the fourth exon of the full-length open reading frame serves as a nucleus localization signal and plays a role in interactions with RUBY1. TT8 alone or bound to RUBY1 and WD40 passes through the nuclear membrane and forms a complex at the nucleus. Subsequently, a regulatory complex formed by TT8, RUBY1, and WD40 directly transactivates expression of flavonoid 3′-hydroxylase (*F3*′*H*) to synthesize anthocyanin. Simultaneously, another regulatory complex comprising TT8 and an MYB5-type partner directly transactivates expression of a proton pumping gene (*PH8*) to induce vacuole acidification for anthocyanin transport. Consequently, it can be inferred that fruit quality may be promoted by two regulatory complexes formed by TT8 with different MYB-type partners.

This study highlights the importance of alternative splicing of *TT8* effectively improving multiple fruit quality traits in blood orange at the fruit ripening stage. Besides, the mutant fruit lacking pigment in pulp tissues displayed significantly higher expression levels of Δ15-*TT8* in juice sac cells compared with the wild type. Furthermore, this mutant also showed downregulation of enzymatic genes involved in the anthocyanin biosynthetic pathway, resulting in reduction of anthocyanin metabolites in its fruits. In this mutant fruit, the anthocyanin content decreased to 0.35 mg/l at the ripening stage, which was significantly lower than that in the wild type. Moreover, the higher expression of Δ15-TT8 in juice sac cells from the mutant led to several physiological changes, including reduced production of citrate and sugar. Additionally, mRNA levels of Δ15-TT8 in the yellow part of the peel tissues from a chimera fruit were significantly higher than those in the red part (*P* < .05), resulting in lower pigment accumulation in the yellow part of the peel tissues.

In *Petunia hybrida*, bHLH AN1 activates anthocyanin synthesis and acidification of the vacuole in petal cells through interactions with distinct MYB proteins [8]. Intriguingly, bHLH TT8 bound to different MYB-type partners is capable of transactivating expression of *F3′H* and *PH8* to independently promote anthocyanin production and vacuole acidification in orange fruit. However, Δ15-TT8 loses its nuclear localization due to lack of a conserved domain with the SAHIQ motif, resulting in impairment of its ability to transactivate gene expression. Alternative splicing can modulate gene function by adding or removing certain protein domains, and can therefore affect the activity of the protein.

The steep proton gradient across the vacuolar membrane (tonoplast) drives massive transport of citrate into the vacuole [30]. *Citrus* plants have PH5-like homologs that are involved in citrate accumulation; *CsPH8* was predominantly expressed in fruit juice sacs and *CsPH8* seems likely to regulate citrate accumulation in the *Citrus* fruit vacuole [31]. Interaction between CitMYB52 and CitbHLH2 could synergistically transactivate CitALMT (an aluminum-activated malate transporter) to negatively regulate citrate accumulation in *Citrus reticulata* cv. ‘Ponkan’ [32]. In this study, dual-luciferase assays revealed that TT8 interacted synergistically with an MYB5-type partner to regulate expression of a plasma membrane H^+^-ATPase (*PH8*), which positively accumulated citrate in vacuoles by proton gradients. Furthermore, a separate regulatory complex formed by TT8, RUBY1, and WD40 concurrently trans-activates *F3′H*, resulting in the production of anthocyanin. Collectively, these findings highlight the crucial role of TT8 in regulating diverse fruit quality-related metabolic pathways by interacting with a specific MYB-type partner in sweet orange.

ABA also plays pivotal roles in fruit development and ripening. A small-molecule ABA analogue (AM1) was instilled into the main stem of orange trees, which significantly promoted citrus fruit coloring, chlorophyll degradation, and titratable acidity accumulation [24]. Exogenous ABA triggered relevant transcriptional changes and repressed the protein ubiquitination process in sweet orange [33]. Endogenous ABA contents were measured during development and ripening in the wild type and mutant lacking pigment in pulp tissues. ABA content was significantly higher in wild-type fruit (0.35 μg/g FW) on 7 November before fruit ripening compared with mutant fruit (0.29 μg/g, data not shown). However, ABA accumulation in the wild type was constant at 0.37 μg/g FW but ABA in the mutant was 0.32 μg/g FW at the ripening stage, although it was not significantly different between the two genotypes at the ripening stage (*P* > .05). Besides, fruit produced more anthocyanin after exogenous ABA was applied to the fruit tree before ripening. It is possible to hypothesize that ABA could play an important role in regulating anthocyanin biosynthesis before fruit ripening. Based on the results obtained in the transient overexpression assay in this study, the MBW regulatory complex also activates the ABA biosynthetic pathway. However, the interplay mechanism between ABA accumulation and *TT8* expression in blood orange fruit is still unclear.

### Conclusions

The findings of this study reveal that TT8 plays a crucial role in regulating anthocyanin biosynthesis and vacuole acidification by controlling the transcription of downstream genes through two regulatory complexes formed by TT8 and its different MYB-type partners. Moreover, this investigation suggests that increasing mRNA levels of alternative splicing transcripts may function through a negative feedback mechanism to downregulate TT8 expression and then limit anthocyanin accumulation in blood orange fruit.

## Materials and methods

### Characterization of alternative splicing of *TT8*

Blood orange (*C. sinensis* cv. ‘Tarocco’) fruits were harvested at the ripening stage. The pulp or peel tissues of fruits were quickly frozen in liquid nitrogen and stored at −80°C in the laboratory. RNA from juice sac cells of each fruit was extracted using an RNA isolation kit (Huayueyang, Beijing, China). cDNA synthesis was then performed using the PrimeScript First-Strand cDNA Synthesis Kit (Takara, Dalian, China). The amplified products of alternative-spliced *CsTT8* were cloned into a TA cloning vector using a pair of specific primers [4] (Supplementary Data Table S1). Random clones from amplified products for *CsTT8* had been mentioned previously were selected for sequencing to screen for alternative splicing transcripts. As a result, three different alternative splicing events in *TT8*, which result in differing transcript lengths, were identified.

Moreover, in the Peking University Transcription Factor Database (http://planttfdb.gao-lab.org/index.php), 194 transcription factors with a bHLH-type conserved domain were identified in sweet orange ‘Valencia’. The software MegaAlign (7.1.0) was used to perform multiple alignment using different bHLH-type proteins derived from different fruit trees and TT8 from ‘Tarocco’. A phylogenetic tree was constructed for these transcription factors using the neighbor-joining method, with bootstrap calculations performed 1000 times using MEGA7.

### Real-time quantitative PCR

Juice sac cells were harvested from ‘Tarocco’ and its mutant fruit lacking sufficient pigment in pulp tissues at the fruit ripening stage in a commercial orchard in Ziyang city, southwestern China. Additionally, a chimera fruit lacking enough pigment in half of the peel tissue was selected at the same orchard by chance to study genes expressed differentially between the different parts of the peel tissues.

The total RNAs of pulp or peel tissues were isolated using an RNA isolation kit (Huayueyang, Beijing, China). cDNA synthesis was performed as described previously. Aliquots of 50 μl cDNA were diluted to 100 μl with ddH_2_O and 2 μl of template was used for real-time PCR on an LC480 instrument (Roche, Basel, Switzerland). The relative quantification of transcript was calculated the using 2^−△△Ct^ method. The PCR program consisted of an initial denaturation at 95°C for 3 min, followed by 35 cycles of denaturation at 95°C for 15 s, annealing at 56°C for 15 s, and extension at 72°C for 20 s, with a final extension at 72°C for 2 min. All pairs of primers used in this study are listed in Supplementary Data Table S1. The relative expressions (fold) of genes between wild type and each mutant lacking pigment in pulp or peel tissues were normalized, and the reference gene *EF-1α* was used as the internal standard.

### Fruit quality analysis

Fruit juice was extracted using a domestic juicer. The titratable acidity and soluble sugar contents were determined using previously described methods [4]. The anthocyanin content of fruit juice was measured at 510 nm by spectrophotometry as described previously [18]. Additionally, citrate in pulp tissues from different samples at the fruit ripening stage was determined using HPLC-MS/MS as reported in a previous study [19]. Moreover, the concentrations of plant ABA or IAA in juice sac cells from pulp tissues were measured using an ELISA kit (Sino-Best, Shanghai, China).

### Yeast one-hybrid and yeast two-hybrid assays

The yeast one-hybrid assay was performed to investigate interaction capacities of RUBY1, WD40, TT8 (or truncated TT8 proteins) with the promoter region from *flavonoid 3′-hydroxylase* (*F3′H*), which is involved in anthocyanin biosynthesis (Supplementary Data Table S1). The empty pAbAi bait vector was digested by the double enzyme SalI and SmaI (Takara, Japan). Subsequently, the amplified promoter region (pCsF3′H) was fused into the pAbAi bait vector using homologous recombination with the In-Fusion HD Enzyme kit (Clontech, Japan) to generate pAbAi-pF3′H. Simultaneously, the full-length open reading frame of *TT8*, *RUBY1*, and *WD40* and the partial length of Δ − *TT8* were fused into pGADT7 prey vector using homologous recombination with the In-Fusion HD Enzyme Kit (Clontech, Japan) to construct pGADT7-TT8, pGADT7-RUBY1, pGADT7-WD40, pGADT7-Δ15-TT8, and pGADT7-ΔC200-TT8. Bait and prey constructs were co-transformed into Y1HGold yeast cells. The yeast cells were selected on SD-Leu medium supplemented with 100 μM AbA.

To test the yeast two-hybrid between TT8 (or truncated TT8 proteins) and RUBY1 and WD40, the full-length or partial-length open reading frames of *TT8* were fused into pGBKT7 BD vector using homologous recombination with the In-Fusion HD Enzyme Kit (TAKARA, Japan) to generate pGBKT7-TT8, pGBKT7-Δ15-TT8, pGBKT7-ΔN132-TT8, pGBKT7-ΔN200-TT8, pGBKT7-ΔC690-TT8, pGBKT7-ΔC649-TT8, pGBKT7-△C567-TT8, pGBKT7-△C537-TT8, and pGBKT7-ΔC207-TT8. Simultaneously, the full-length open reading frames of TT8, RUBY1, and WD40 were fused into pGADT7 AD vector using homologous recombination with the In-Fusion HD Enzyme Kit (Clontech, Japan) to generate pGADT7-TT8, pGADT7-RUBY1, and pGADT7-WD40, respectively. Therefore, BD and AD recombinant vectors fused with proteins of interest, as well as the empty pGBKT7 (or pGADT7), were co-transformed into AH109 yeast cells. The yeast cells co-transformed by different combination of BD and AD vectors were grown on SD-Leu-Trp, SD-Leu-Trp-His-Ade, and SD-Leu-Trp-His-Ade adding X-α-Gal media respectively to analyze *in vitro* interactions between different proteins.

### Subcellular localization of transcription factors

In this study, we amplified the full-length open reading frame for *TT8*, *RUBY1*, and *WD40* and alternative splicing transcript Δ15-*TT8* lacking the fourth exon using different pairs of primers (Supplementary Data Table S1), and each amplified fragment was then fused into a vector of p35S1300-EGFP using ClonExpress II One Step Cloning Kit (Vazyme, China) via homologous recombination. The resulting constructs, namely p35S1300-EGFP-TT8, p35S1300-EGFP-Δ15-TT8, p35S1300-EGFP-RUBY1, and p35S1300-EGFP-WD40, were then transformed into the competent *Agrobacterium* GV3101. In the agroinfiltration assay, *Agrobacterium* cultures transformed by each construct were mixed prior to infiltration into leaves of *N. benthamiana*. In the preliminary subcellular localization experiment, the epidermis of inoculated leaves was stained by DAPI before observation using a laser confocal microscope (LSM800, Carl Zeiss, Germany).

Subsequently, mesophyll protoplasts from *A. thaliana* were prepared for nucleus co-localization experiments using a nuclear-localized marker and different constructs fused with the protein of interest. The open reading frames of *TT8*, Δ15-*TT8*, and *WD40* were amplified using pairs of primers, and were then inserted into pFGC5941-p35S-GFP to generate pFGC5941-GFP-TT8, pFGC5941-GFP-Δ15-TT8, and pFGC5941-GFP-WD40, respectively. A polypeptide with MDPKKKRKV at amino acid level was used as a nuclear-localized marker. Protoplasts were extracted from *A. thaliana* and then transformed with each construct using the polyethylene glycol (PEG) method [20]. The fluorescence signal of transient expression from a fused GFP-protein of interest was observed using a confocal scanning microscope (C2-ER, Nikon, Japan).

### Dual luciferase reporter assay

Pairs of primers (Supplementary Data Table S1) were designed by online software (https://crm.vazyme.com/cetool/singlefragment.html). Each coding sequence of *TT8*, Δ15-*TT8*, *RUBY1*, *WD40*, and *PH4* from blood orange, *MYB5* from navel orange, and the promoter region of *F3′H* and *PH8* from blood orange were amplified using PrimeSTAR Max DNA Polymerase (Takara, Japan). Subsequently, pGreen-SK-62 was digested using double enzymes BamHIand SalI(Takara, Japan). The resulting pGreen-SK-62 and PCR-amplified products for each gene of interest were fused by homologous recombination to generate an effector vector using the ClonExpress II One Step Cloning Kit (Vazyme, Nanjing, China). Additionally, BamHIand Hind III (Takara, Japan) were used to digest pGreen-0800, then the above-mentioned pGreen-0800 and PCR amplified product for each promoter region was fused by homologous recombination to generate a reporter vector using the ClonExpress II One Step Cloning Kit (Vazyme, Nanjing, China). Each effector and reporter construct was co-transformed into *Agrobacterium* GV3101.

The effector (or reporter) strains were cultured and then resuspended with infiltration buffer (10 mM MES, 10 mM MgCl_2_, 200 mM acetosyringone, pH 5.6) to OD 600 at 1.0–1.5. The effector and reporter cultures were mixed together and infiltrated onto *N. benthamiana* leaves with needleless syringes [21]. Firefly luciferase (LUC) and *Renilla* luciferase (REN) activities were measured 3 days after infiltration using the Dual Luciferase Reporter Assay Kit (Vazyme, Nanjing, China). The transcription activation activity of TT8 and others on the *F3′H* (or *PH8*) promoter was calculated using the LUC/REN ratio. At least five biological replicates were performed for each combination of different effectors.

### Transient overexpression in tobacco

*TT8*, Δ15-*TT8*, *RUBY1*, and *WD40* from ‘Tarocco’ fruit were amplified using PrimeSTAR Max DNA Polymerase (Takara, Japan). The empty pBI121 vector was linearized through double enzymatic digestion using BamHIand SacI simultaneously. Afterwards, the amplified products for *TT8*, Δ15-*TT8*, *RUBY1*, and *WD40* were fused with linearized pBI121 vector by homologous recombination using the ClonExpress II One Step Cloning Kit (Vazyme, China) to generate pBI121-TT8, pBI-Δ15-TT8, pBI-RUBY1, and pBI-WD40, respectively, using specific pairs of primers (Supplementary Data Table S1). Consequently, overexpression vectors were transformed into *Agrobacterium* GV3101. *Agrobacterium*-mediated transient expression in leaves of *N. benthamiana* were performed to verify the regulation function of TT8 alone or in combinations of TT8 and its partners [22]. After agroinfiltration for 4 days, the inoculated leaves were harvested to study the relative expression levels of genes, total anthocyanin, citrate, and ABA contents, measured using methods mentioned above.

### Exogenous ABA spraying of blood orange trees

Exogenous application of 200 μM ABA to ‘Tarocco’ trees was carried out on 20 November at the same orchard. The same trees were sprayed again with exogenous ABA at 200 μM on 19 December. The ripening fruits were harvested on 17 March in the next year. Juice sac cells from pulp tissues were frozen in liquid nitrogen and stored at −80°C in the laboratory. Fruit juice was extracted using a domestic juicer. The total anthocyanin contents, titratable acidity, total soluble sugars, and relative expression levels of *TT8* were measured in comparison with ABA-treated and water control samples using previously mentioned protocols.

### Statistical analysis

Statistical differences in fruit quality between the genotypes were evaluated using ANOVA by SPSS (IBM, USA). Statistical significance was considered as *P* < .05.

## Acknowledgements

This work was supported by Sichuan Province International Science and Technology Innovation and Cooperation Program (22GJHZ0183) and the Chengdu Municipality Technology Innovation and Development Program (2022-YF05-00450-SN).

## Author contributions

W.J.H. and X.R. were responsible for identification of alternative splicing and transcripts. X.R., Q.S.P., W.W.C., and Z.F. carried out qPCR, yeast hybrid, subcellular localization, transient overexpression, and fruit quality experiments. W.J.H. conceived the experimental design and drafted the manuscript. W.J.H. proposed and supervised the research. All authors read and approved the final manuscript.

## Data availability statement

Three transcription factors, WD40 (GenBank accession number KT757349), RUBY1 (KT757348), and TT8 (KT757350), from blood orange ‘Tarocco’ have been deposited in GenBank. The sequences of a total of 194 bHLH with conserved motif of DNA­-binding superfamily proteins selected from ‘Valencia’ at the ICGC database in Peking have been deposited in the University Transcription Factor Database (http://planttfdb.gao-lab.org/index.php).

## Conflict of interest

The authors declare that they have no conflict of interest.

## Supplementary Material

Web_Material_uhad190
